# Multi-Gene Genetic Programming-Based Identification of a Dynamic Prediction Model of an Overhead Traveling Crane

**DOI:** 10.3390/s22010339

**Published:** 2022-01-03

**Authors:** Tom Kusznir, Jaroslaw Smoczek

**Affiliations:** Department of Manufacturing Systems, Faculty of Mechanical Engineering and Robotics, AGH University of Science and Technology, al. Mickiewicza 30, 30-059 Kraków, Poland; smoczek@agh.edu.pl

**Keywords:** nonlinear identification, genetic programming, crane

## Abstract

This paper proposes a multi-gene genetic programming (MGGP) approach to identifying the dynamic prediction model for an overhead crane. The proposed method does not rely on expert knowledge of the system and therefore does not require a compromise between accuracy and complex, time-consuming modeling of nonlinear dynamics. MGGP is a multi-objective optimization problem, and both the mean square error (MSE) over the entire prediction horizon as well as the function complexity are minimized. In order to minimize the MSE an initial estimate of the gene weights is obtained by using the least squares approach, after which the Levenberg–Marquardt algorithm is used to find the local optimum for a *k*-step ahead predictor. The method was tested on both a simulation model obtained from the Euler–Lagrange equation with friction and the experimental stand. The simulation and the experimental stand were trained with varying control inputs, rope lengths and payload masses. The resulting predictor model was then validated on a testing set, and the results show the effectiveness of the proposed method.

## 1. Introduction

Material handling systems such as overhead cranes are widely used in the industrial sector for the transportation of heavy or hazardous loads over short distances. Since transient oscillations can be dangerous to both the surroundings and the payload itself, an effective dampening of oscillations has become a focus of research. Effective positioning, as well as residual oscillations, decrease the time spent on loading and unloading [1]. As an underactuated mechanical system, the sway cannot be arbitrarily controlled, requiring a need for the development of effective control strategies. Model-based control systems, such as model predictive control, utilize mathematical mapping between the input and output of the system in order to take advantage of the system dynamics.

A thorough review of various methods for crane dynamic modeling and control reported in the literature up to 2001 is presented in [2], while more current, state-of-the-art methods (up to 2016) are discussed in [3]; however, most of the presented approaches are derived from the Euler–Lagrange equation. Other methods that have been applied to modeling material handling systems include Takagi–Sugeno fuzzy models [4,5], bond graph methods [6], multi-body dynamics [1,7] and neural networks [8]. Evolutionary algorithms have been implemented in a variety of crane applications including anti-sway crane control [9], scheduling [10,11] and proactive maintenance [12].

The method proposed in this paper follows the previous work [13], in which the MGGP with least square parameter estimation was applied to identify the direct *k*-step ahead predictors of the crane dynamics by training and validating them with data sets obtained from simulations carried out on a model derived from the Euler-Lagrange equation. The direct prediction-based method developed in [13] requires *n* different models to predict over the prediction horizon *n*. This approach is less prone to bias compared to the iterated prediction method when there is model misspecification; however, the large number of models required reduces the interpretability of the output dynamics, which is a significant advantage of GP.

In this paper MGGP-based identification is proposed for an iterative prediction of crane dynamics. Two separate sets of input–output data were obtained. The first is from a simulated model developed by the Lagrange approach and the second from an experimental stand. To develop the iterated *k*-step ahead predictor the cost function to be minimized was the mean square error over the whole prediction horizon, leading to a nonlinear least square estimation for the gene weights. The individual gene weights were initially estimated using the least squares approach, after which the Levenberg–Marquardt algorithm was used to find the local optimum. 

The contributions of this paper are as follows:

- This study uses data from both a simulated model as well as from the experimental stand for obtaining an approximating function to map the input to the output using multi-gene genetic programming. To the best of the authors’ knowledge this is the first study that uses genetic programming for an iterative predictive model of the crane dynamics.
- The Levenberg–Marquardt algorithm is used for minimizing the square errors over the entire prediction horizon using the least squares from the standard MGGP algorithm as an initial point.

The paper is organized as follows: In Section 2 an overview of genetic programming for the use of system identification is presented along with the theoretical background to MGGP and the nonlinear least square estimation of the parameters of the iterative prediction model using the Levenberg–Marquardt algorithm. In Section 3 the proposed method is validated on a simulation model in which the crane dynamics are developed using the Euler–Lagrange equation. The proposed method is then validated on the experimental setup in Section 4. The concluding remarks are made in Section 5.

## 2. Technical Background

Nonlinear modelling can be divided into three broad categories: white-box modeling, in which the models are derived purely from first principles; black-box modeling, in which the model is derived from measurement data and no expert knowledge of the system is required; and gray-box modeling, which is a combination of the previous two, in that some knowledge of the system can be incorporated into the modeling process. The nonlinear identification of complex systems based on first principles can be costly and imprecise due to incorrect assumptions resulting in plant–model mismatch. The NARMAX (Nonlinear autoregressive moving average with exogenous variables) approach to nonlinear system identification is of particular interest as it is capable of modeling a significant portion of nonlinear systems [14,15].

Genetic programming was developed by Koza [16] as an evolutionary algorithm that can synthesize programs. The ability to synthesize programs gives it an advantage over other black-box modeling methods in that the developed model is more interpretable for future applications. In [17] a linear-in-the-parameter model was developed using genetic programming based on the Gabor–Kolmogorov analysis of variance decomposition. In [18] a STROGANOFF (structured representation on genetic algorithms for nonlinear function fitting) was developed to overcome difficulties in traditional genetic programming for identification using a local hill-climbing approach. Multi-gene genetic programming [19] was proposed to evolve a sequence of functions with weights estimated by the least squares method. Genetic programming for symbolic regression using a nonlinear least squares method for parameter estimation was studied in [20] and shown to improve performance on a wide array of symbolic regression problems. Applications of genetic programming in the development of prediction models include [21] developing a model predictive control based on a model identified by genetic programming; [22] the identification of a prediction model for the time dependent total creep in concrete; and the development of transient storage models [23].

The genetic program searches both the model structure as well as the parameter space. At the beginning a set of individuals, each representing a candidate solution, are generated to form a population. When using genetic programs to obtain prediction models each individual maps the input to the output by some approximating function f:I→O and is assigned a fitness value [24]. 

**Figure 1:**
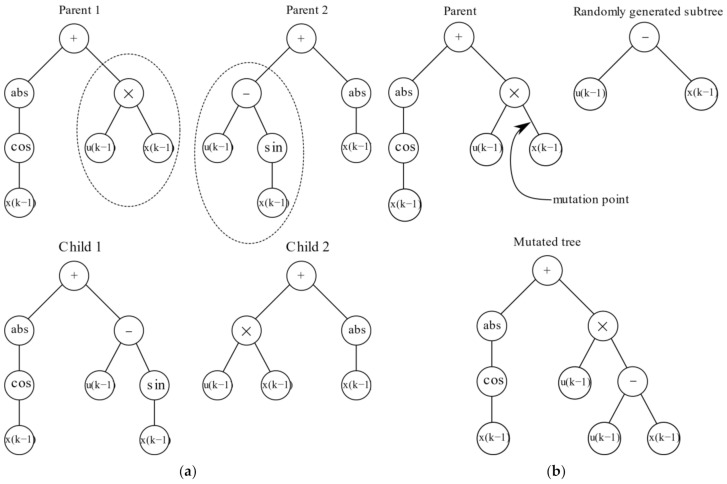
Genetic operators: (**a**) crossover (**b**) mutation.

Several genetic operators are used to evolve each generation to find the optimum solution within the search space. The first is the selection operator, which obtains a subpopulation, $P^{\prime }\subseteq P$, where $n=\left|P^{\prime }\right|$ is the number of selected individuals [24,25]. The other common operators are crossover and mutation. Once parents are selected from the population, subtree crossover selects a point on both parents which will be exchanged. Care must be taken so that the arity of the selection points are the same. An example of subtree crossover is shown in Figure 1a. The mutation operator selects an individual and a tree node is selected at random, a new tree is generated with a depth no greater than the difference of the maximum allowable depth and the depth of the randomly selected node. An example is shown in Figure 1b.

### 2.1. Multi-Gene Genetic Programming

**Figure 2:**
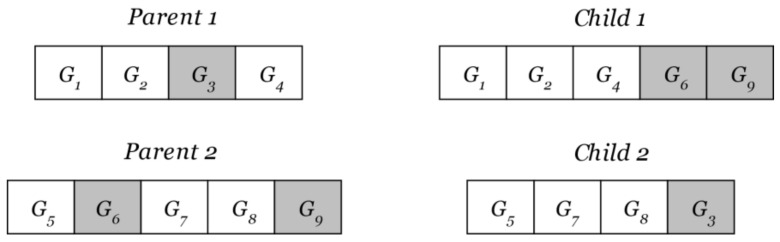
High-level crossover operation.

Multi-gene genetic programming is a genetic programming technique in which the approximating function is composed of a weighted sum of the genes. An additional high-level crossover operation is present in MGGP. The genes of an individual can be swapped with the genes of another individual as long as the maximum number of genes is not exceeded. The high-level crossover operation is shown in Figure 2.

The genes are nonlinear functions obtained by

(1) $$\hat{y}\left(\theta ,x\right)=\theta_{0}+\overset{i=n}{\underset{i=1}{\sum }}\theta_{i}G_{i}\left(x\right)$$

where *θ*_0_ is the bias term, *θ_i_* is the *i*-th gene weight and the vector of outputs is obtained by evaluating the tree-based structure. The least squares method is used to obtain the linear-in-the-parameter gene weights.

(2) $$\theta ={\left(G^{T}G\right)}^{-1}G^{T}y$$

### 2.2. Nonlinear Parameter Estimation

The one-step-ahead linear-in-the-parameters model obtained using multi-gene genetic programming described previously has a NARX model structure, which can be described by

(3) $$y\left(k\right)=f\left(u\left(k-1\right),\ldots ,u\left(k-n_{a}\right),y\left(k-1\right),\ldots ,y\left(k-n_{b}\right)\right)+e\left(k\right)$$

**Figure 3:**
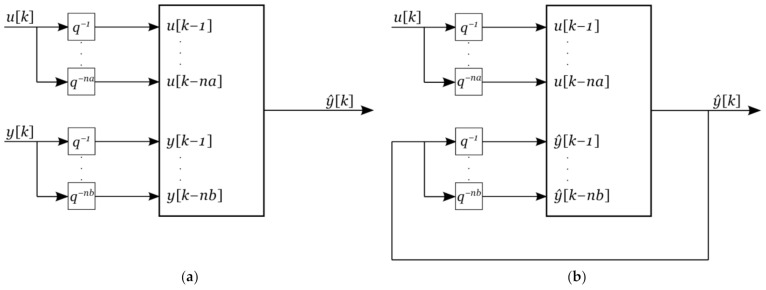
Model structure: (**a**) equation error model; (**b**) output error model.

The model structure described in (3) is an equation error model shown in Figure 3a. For the *k*-step ahead predictor, (3) is iterated *k* times by closing the loop and using simulated values as our new inputs, as is shown in Figure 3b. The closed loop system turns the parameter estimation problem into a nonlinear optimization problem.

The objective function is taken as the MSE given by (4).

(4) $$J=\frac{\sum_{k=1}^{P}\sum_{\max\left(n_{a},n_{b}\right)+1}^{N-P}{\left({\hat{y}}_{k+i}-y_{k+i}\right)}^{2}}{\left(N-P-\max\left(n_{a},n_{b}\right)\right)P}$$

The objective function (4) is a nonlinear least squares problem and can be minimized by using a quasi-Newton approach [26]. The Jacobian of the objective function is obtained by using the finite difference approach. To avoid the computation of many second order derivatives, the Hessian is approximated by $H=J^{T}J$. This creates a positive definite and symmetric matrix and is a reasonable approximation as long as the residuals remain small.

Once the gradient and Hessian are obtained the Levenberg–Marquardt algorithm is used to find the parameters θ according to

(5) $$\theta_{k}=\theta_{k-1}-h_{n-1}{\left(J_{k-1}{}^{T}J_{k-1}+\lambda_{k-1}I\right)}^{-1}J_{k-1}^{T}f_{k-1}$$

where the parameter λ are updated using the algorithm in [27].

## 3. Crane Dynamic Prediction—A Simulation Study

### 3.1. Crane Dynamics

**Figure 4:**
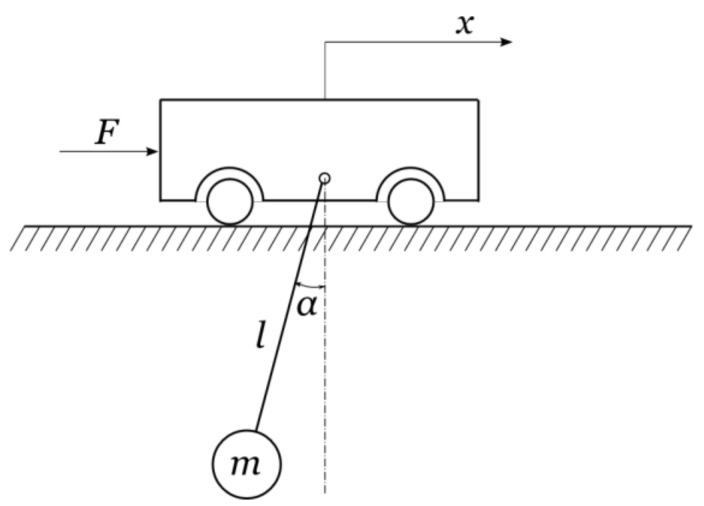
Planar model of an overhead crane.

Figure 4 illustrates a planar model of an overhead crane, which consists of a trolley of mass *M*, moving along a fixed bridge only in the *x*-axis direction. A payload, of mass *m*, is attached to the trolley by a hoisting rope of length *l*. Furthermore, the payload is treated as a point mass, and the hoisting rope is considered to be inflexible and does not undergo any deformation.

The dynamic equations of the overhead crane are obtained using the Euler–Lagrange equation.

(6) $$\frac{d}{dt}\left(\frac{\partial L}{\partial \dot{q}}\right)-\frac{\partial L}{\partial q}=F$$

The generalized coordinates and generalized forces are *q* = [*x*,*α*]*^T^* and *F*, respectively. The equations of motion are given by

(7) $$\left(M+m\right)\ddot{x}+ml\ddot{\alpha }\cos\alpha -ml{\dot{\alpha }}^{2}\sin\alpha =F\;\;$$

(8) $$ml^{2}\ddot{\alpha }+ml\cos\alpha \ddot{x}+mgl\sin\alpha =0\;\;$$

The generalized force *F* consists of both the actuating force produced by the crane motors as well as the friction forces acting between the trolley and the bridge. Motivated by the crane dynamic model with nonlinear friction presented in [28,29], the friction force is given by

(9) $$F_{fr}=f_{fr0}\tanh\left(\frac{\dot{x}}{\xi }\right)+k_{p}\dot{x}+k_{r}\left|\dot{x}\right|\dot{x}$$

where the first term corresponds to a smoothed Coulomb friction model, and the second term corresponds to viscous damping between the trolley and the bridge, while the last term approximates other nonlinear effects. The friction-related parameters are $\xi ,\;f_{fr0},\;k_{p},\;k_{r}\in {\mathbb{R}}^{1}$.

### 3.2. Simulation Results

The training and testing data were obtained from the simulation model (7) and (8) for the parameters given in Table 1, different rope lengths and payload masses varying within the range 10–50 kg and 0.5–3 m, band-limited Gaussian white noise input excitation signal with zero mean and sampling time *t_s_* = 0.1 s.

A multi-objective MGGP, based on the NSGA-II algorithm [30], was used to find the rank and crowding distance of the individual, which was then used for selection. The objectives were to find a model that minimized the prediction error over the whole prediction horizon as well as the model complexity. The complexity was obtained by summing the nodes in each gene of the individual. The MGGP parameters for the simulation study are given in Table 2. Since the Levenberg–Marquardt algorithm relies on an approximation of the Hessian that is satisfied when the residuals are small, the functions that have a large one-step ahead residual do not proceed to optimizing the parameters with the nonlinear least squares. This also reduces the computational complexity of the proposed method.

The analytic quotient [32] is given in (10)

(10) $$aq\left(a,b\right)=\frac{a}{\sqrt{b^{2}+0.0001}}$$

The statistical performance methods used in this article to determine the performance of the predictive model were the normalized mean square error (11) and R^2^ (12).

(11) $$NMSE=\frac{\sqrt{\frac{1}{n+1}{\left(y-\hat{y}\right)}^{2}}}{\sqrt{\frac{1}{n+1}{\hat{y}}^{2}}+\sqrt{\frac{1}{n+1}y^{2}}}$$

(12) $$R^{2}=1-\frac{{\left(y-\hat{y}\right)}^{2}}{{\left(y-\bar{y}\right)}^{2}}$$

The proposed method was compared with an ARX model with two lags for both the input and output as given in (13) and (14). This corresponds to a second-order system that is widely used in crane dynamic modeling [2,3]. The coefficients of the ARX model were obtained by using the least squares method.

(13) $$x\left(k\right)=b_{1}u\left(k-1\right)+b_{2}u\left(k-2\right)+a_{1}x\left(k-1\right)+a_{2}x\left(k-2\right)+e\left(k\right)$$

(14) $$\alpha \left(k\right)=b_{1}u\left(k-1\right)+b_{2}u\left(k-2\right)+a_{1}\alpha \left(k-1\right)+a_{2}\alpha \left(k-2\right)+e\left(k\right)$$

A total of 16 numerical simulations were carried out using different input sequences, rope lengths and payload masses. Out of the 16 simulations, 12 were used for training with the remaining 4 used for testing the performance of the predictor. The testing runs used the same input sequence with varying parameters.

The equations chosen from the Pareto frontier for the position and sway are given in (15) and (16), respectively, while the weights of the genes are presented in Table 3. The coefficients of the ARX model are presented in Table 4.

(15) $$x\left(k\right)=\theta_{1}x\left(k-2\right)+\theta_{2}x\left(k-1\right)+\theta_{3}u\left(k-1\right)+\theta_{4}t_{s}+\theta_{5}u\left(k-2\right)+\theta_{6}aq\left(u\left(k-5\right),x\left(k-3\right)\right)$$

(16) $$\alpha \left(k\right)=\theta_{1}\alpha \left(k-2\right)+\theta_{2}\frac{1}{l}\alpha \left(k-1\right)+\theta_{3}\frac{1}{l}u\left(k-2\right)+\theta_{4}\alpha \left(k-1\right)+\theta_{5}\frac{1}{l}u\left(k-3\right)+\theta_{6}\alpha {\left(k-1\right)}^{2}u\left(k-1\right)\;$$

**Figure 5:**
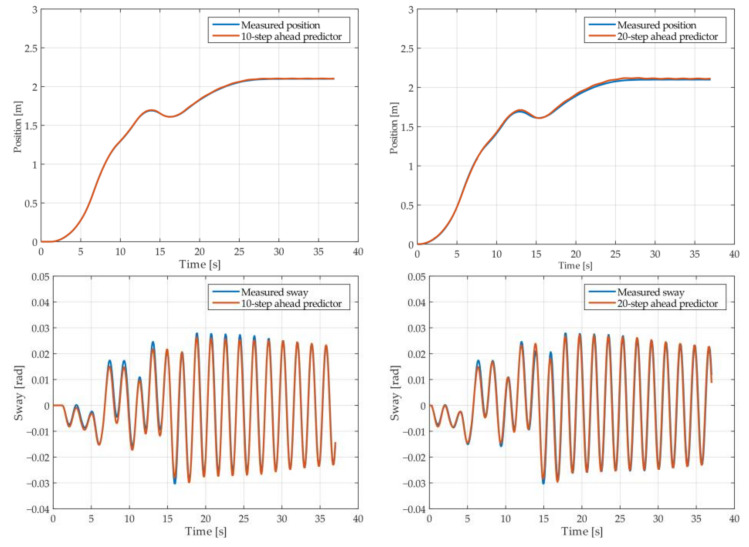
The 10- and 20-step ahead prediction with the MGGP model of the simulated crane position and payload sway with 0.9 m rope length and 10 kg payload mass.

**Figure 6:**
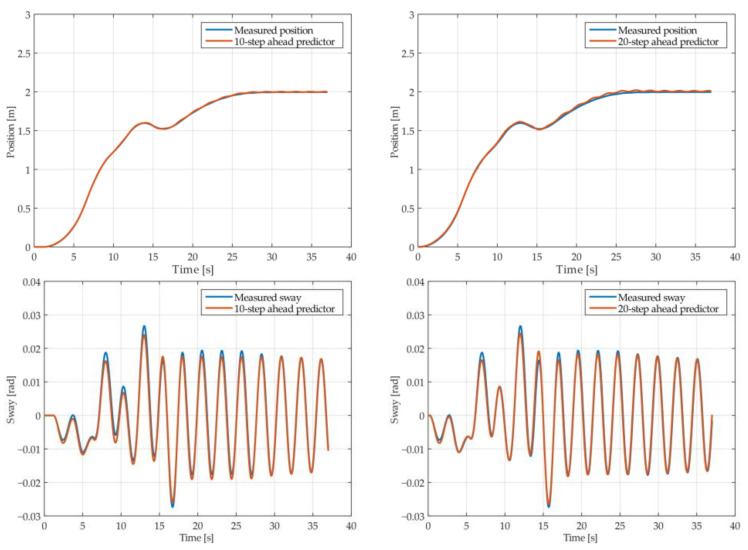
The 10- and 20-step ahead prediction with the MGGP model of the simulated crane position and payload sway with 0.9 m rope length and 40 kg payload mass.

**Figure 7:**
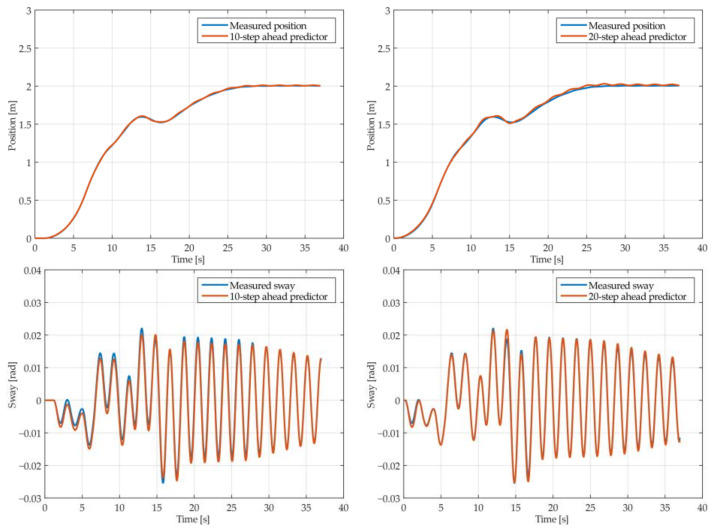
The 10- and 20-step ahead prediction with the MGGP model of the simulated crane position and payload sway with 1.3 m rope length and 40 kg payload mass.

**Figure 8:**
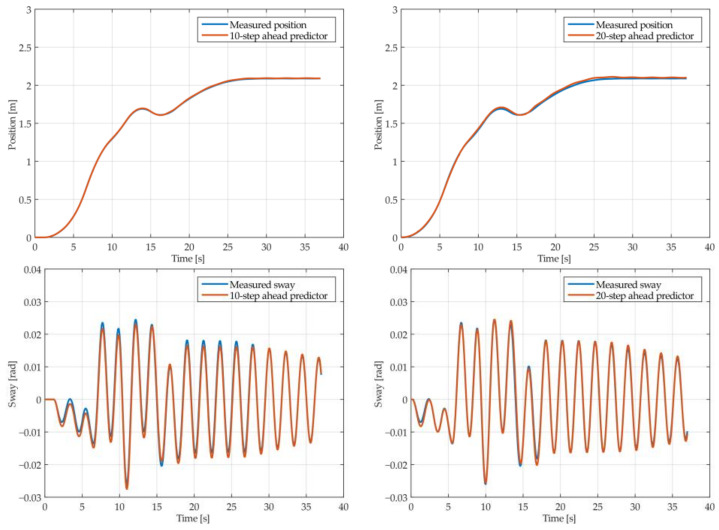
The 10- and 20-step ahead prediction with the MGGP model of the simulated crane position and payload sway with 1.7 m rope length and 10 kg payload mass.

**Figure 9:**
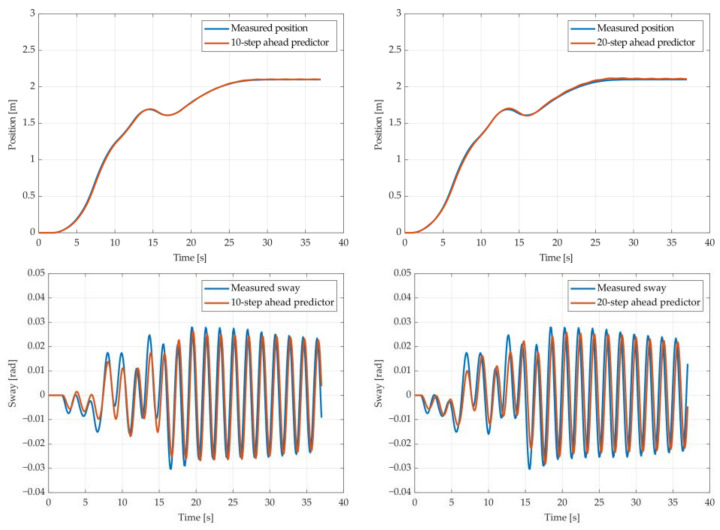
The 10- and 20-step ahead prediction with the ARX model of the simulated crane position and payload sway with 0.9 m rope length and 10 kg payload mass.

**Figure 10:**
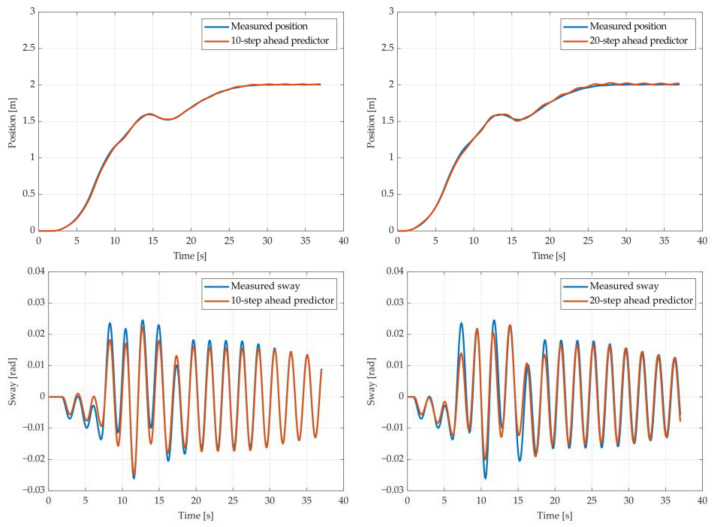
The 10- and 20-step ahead prediction with the ARX model of the simulated crane position and payload sway with 1.3 m rope length and 40 kg payload mass.

The results of the MGGP and ARX models’ 10-step and 20-step ahead prediction of the crane position and payload sway were compared with the testing data obtained from the numerical simulations in Figure 5, Figure 6, Figure 7, Figure 8, Figure 9 and Figure 10, respectively. The normalized mean square error and the R^2^ of the position and sway prediction for the 10-step ahead and 20-step ahead prediction for the MGGP model and ARX model are given in Table 5. We noticed that the position model obtained by the MGGP has a similar structure to the ARX model with an additional nonlinear term. The MGGP model and ARX model accurately predicted the position of the crane with no significant loss of accuracy as the prediction step increased. However, the ARX model was a poor predictor of the payload sway, and there was a significant loss of accuracy as the prediction step was increased. The mean and standard deviation of the NMSE for the MGGP sway model were 0.0586 and 0.0021 for the 10-step ahead predictor and 0.0518 and 0.0209 for the 20-step ahead predictor, while for the ARX sway model they were 0.2387 and 0.0933 for the 10-step ahead predictor and 0.3714 and 0.1514 for the 20-step ahead predictor. The mean and standard deviation of the NMSE for the MGGP position model were 0.0015 and 2.0616 × 10^−4^ for the 10-step ahead predictor and 0.0047 and 1.7321 × 10^−4^ for the 20-step ahead predictor, while for the ARX sway model they were 0.0028 and 5 × 10^−5^ for the 10-step ahead predictor and 0.0044 and 1.4141 × 10^−4^ for the 20-step ahead predictor. 

## 4. Experimental Results

**Figure 11:**
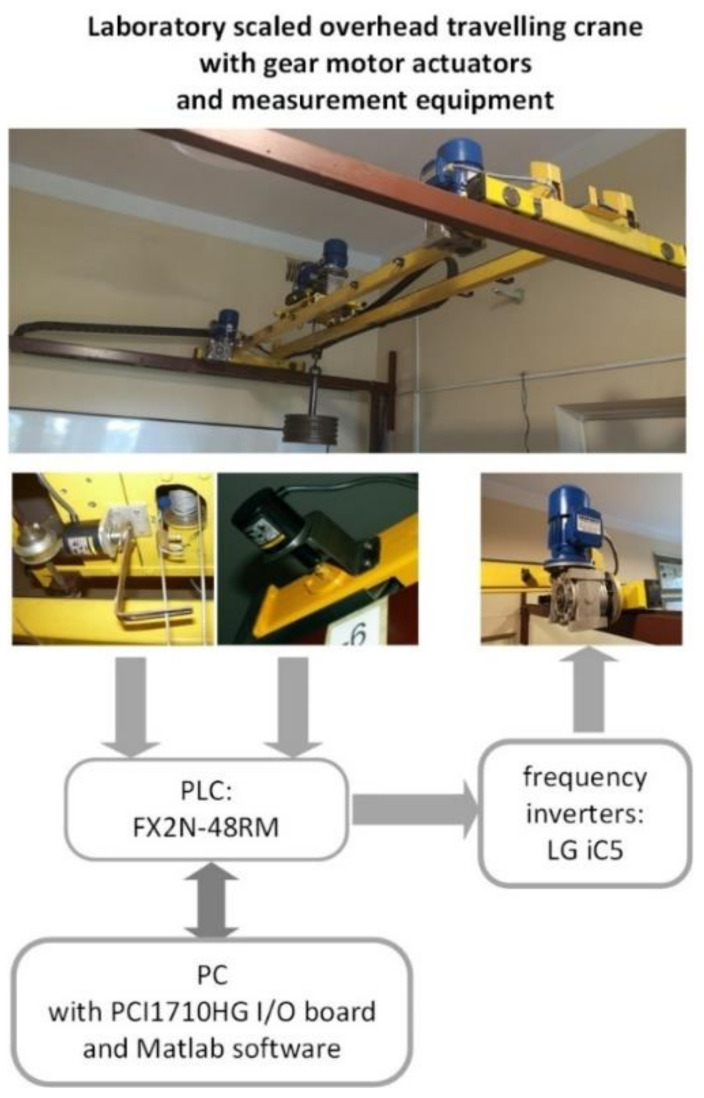
The laboratory stand.

Crane dynamic model identification was based on data measured during experiments carried out on the laboratory-scaled overhead traveling crane presented in Figure 11. The identification was focused on the double girder crane mechanism driven by the two AC gear motors with 0.18 kW output power, 1400 rpm speed and 15.5 gear ratio, which were supplied by the two LG iC5 0.4 kW frequency inverters. The crane system was controlled by using the Mitsubishi FX2N series programmable logic controller (PLC). The crane’s bridge position was measured by using the incremental encoder with a resolution of 400 pulses per rotation (ppr), while the sway angle of a payload suspended on the rope was measured using the encoder with a resolution of 2000 ppr, which was installed under the trolley and connected with fork-bottomed arms embracing the hoisting rope. The experimental data were measured with a sample time of 0.1 s using PC (8GB RAM, CPU Intel Core i7-6700K Quad Core 4GHz, Windows 10) equipped with the I/O board (PCI1710HG) and MATLAB software release R2020.

**Figure 12:**
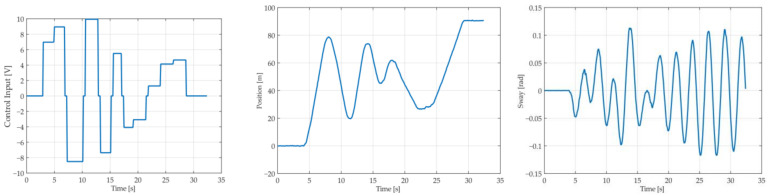
Excitation input signal (control input), position response and sway response for training data set with rope length of 1.2 m and payload mass of 20 kg.

The training data were collected from 12 experiments carried out with different rope lengths in the range of 0.6–2 m and the payload mass in the range of 10–50 kg, while the testing data were obtained from the experiments performed for 0.9-meter, 1.3-meter and 1.7-meter ropes and a payload mass between 10 kg and 40 kg. The experimental input sequence consisted of a series of step functions with varying amplitudes to try to excite the system without violating the input as well as positional constraints. An example of the input sequence with the corresponding position and sway response from one of the training data sets is presented in Figure 12. Table 6 presents the MGGP parameters.

The equations chosen from the Pareto frontier for the position and sway are given in (17) and (18), respectively, and the weights of the genes are presented in Table 7, and the coefficients of the ARX models are presented in Table 8.

(17) $$\begin{array}{c} x\left(k\right)=\theta_{1}x\left(k-2\right)+\theta_{2}u\left(k-6\right)\\ \;+\theta_{3}aq\left(aq\left(u\left(k-8\right),aq\left(u\left(k-9\right)+t_{s},u\left(k-8\right)\right)\right),aq\left(u\left(k-9\right)+t_{s},u\left(k-8\right)\right)\right)\\ \;+\theta_{4}aq\left(u\left(k-9\right),x\left(k-1\right)\right)+\theta_{5}x\left(k-1\right) \end{array}$$

(18) $$\begin{array}{c} \alpha \left(k\right)=\theta_{1}\frac{1}{l}\alpha \left(k-1\right)+\theta_{2}\alpha \left(k-1\right)+\theta_{3}\alpha \left(k-3\right)+\theta_{4}u\left(k-9\right)\\ \;+\theta_{5}\left(aq\left(u\left(k-5\right),u\left(k-6\right)\right)-\alpha \left(k-1\right)+g-aq\left(u\left(k-5\right),\frac{1}{l}u\left(k-6\right)\right)\right)+\theta_{6}u\left(k-6\right) \end{array}$$

**Figure 13:**
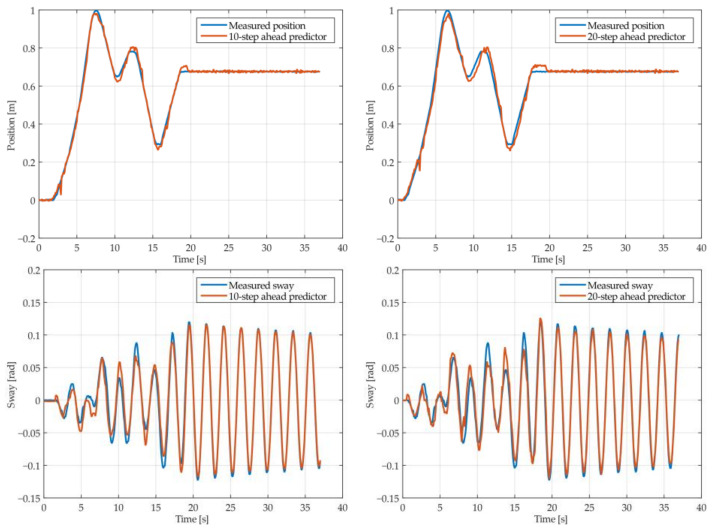
The 10- and 20-step ahead prediction with the MGGP model of the experimental crane position and payload sway with 0.9-meter rope and 10 kg payload mass.

**Figure 14:**
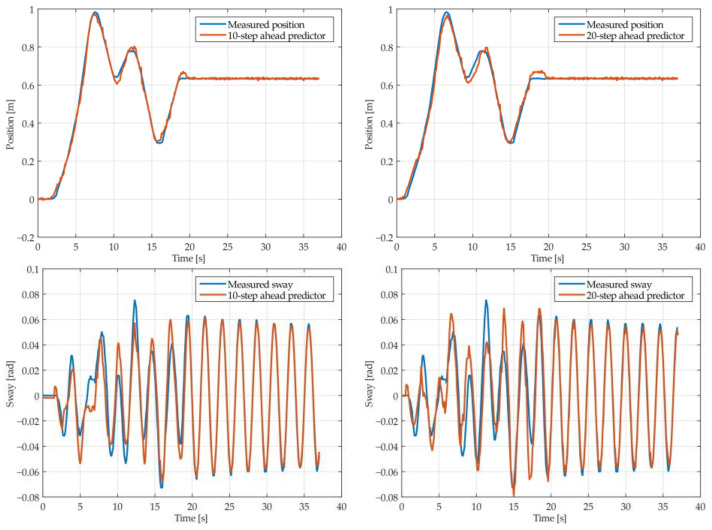
The 10- and 20-step ahead prediction with the MGGP model of the experimental crane position and payload sway with 0.9-meter rope and 40 kg payload mass.

**Figure 15:**
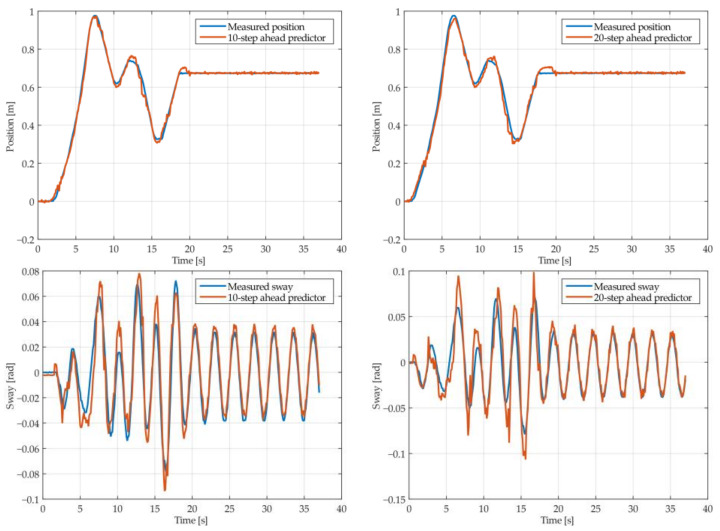
The 10- and 20-step ahead prediction with the MGGP model of the experimental crane position and payload sway with 1.3-meter rope and 40 kg payload mass.

**Figure 16:**
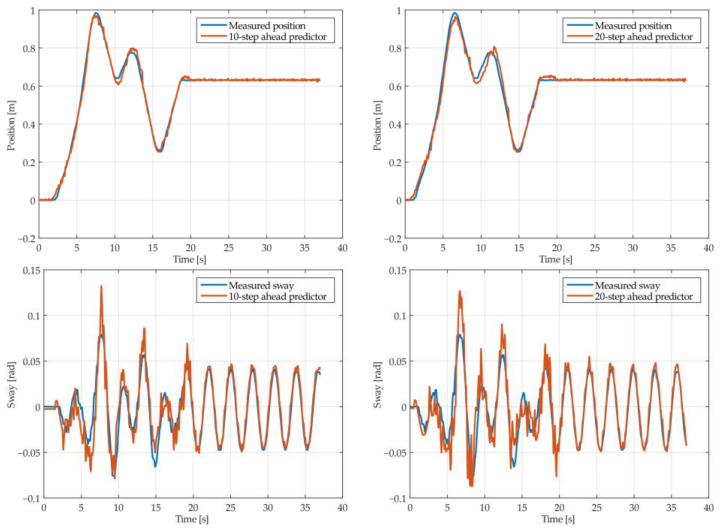
The 10- and 20-step ahead prediction with the MGGP model of the experimental crane position and payload sway with 1.7-meter rope length and 10 kg payload mass.

**Figure 17:**
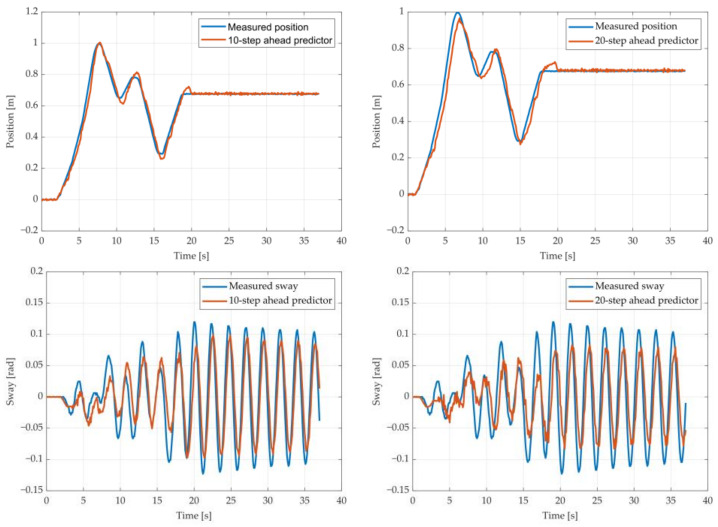
The 10- and 20-step ahead prediction with the ARX model of the experimental crane position and payload sway with 0.9-meter rope and 10 kg payload mass.

**Figure 18:**
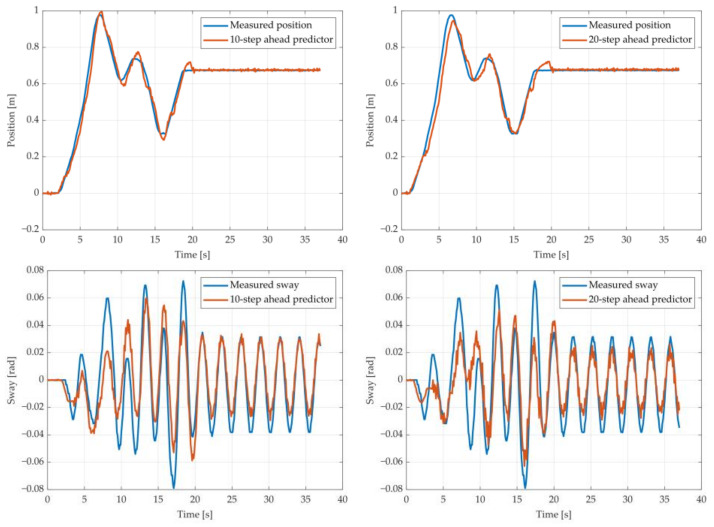
The 10- and 20-step ahead prediction with the ARX model of the experimental crane position and payload sway with 1.3-meter rope and 40 kg payload mass.

The results of the MGGP and ARX models’ 10-step and 20-step ahead predictions of the crane position and payload sway were compared with the testing data obtained from the experimental stand in Figure 13, Figure 14, Figure 15, Figure 16, Figure 17 and Figure 18, respectively. The normalized mean square error and the R^2^ of the position and sway prediction for the 10-step ahead and 20-step ahead predictions for the MGGP model and ARX model are given in Table 9. We can observe from the plots that the MGGP sway model is better at predicting the residual oscillations with the transient oscillations being either over or underpredicted. The ARX sway model either over or underpredicts both the transient as well as the residual oscillations. The mean and standard deviation of the NMSE for the MGGP sway model were 0.1471 and 0.0486 for the 10-step ahead predictor and 0.1519 and 0.0428 for the 20-step ahead predictor, while for the ARX sway model they were 0.3277 and 0.0289 for the 10-step ahead predictor and 0.3984 and 0.0812 for the 20-step ahead predictor. The mean and standard deviation of the NMSE for the MGGP position model were 0.0114 and 4.1633 × 10^−4^ for the 10-step ahead predictor and 0.0167 and 9.6047 × 10^−4^ for the 20-step ahead predictor, while for the ARX sway model they were 0.0224 and 9.6954 × 10^−4^ for the 10-step ahead predictor and 0.0339 and 0.0017 for the 20-step ahead predictor, respectively.

## 5. Conclusions

The paper proposes the use of a multi-gene genetic programming approach for the identification of an overhead crane predictor using input–output data from both a simulated model as well as an experimental stand. Two different types of input data were used for training, Gaussian white noise was used for the simulation, and varying amplitude sequence of step responses were used for the experimental stand. The experiments were carried out at different rope lengths and payload masses. 

The multi-gene genetic programming parameter optimization was performed over the whole prediction horizon *n*. This causes the model to become nonlinear in the parameters in the closed loop. To optimize the parameters, the Levenberg–Marquardt algorithm is used. Both the mean square error over the whole prediction horizon as well as the complexity are objective functions to be reduced; therefore, the NSGA-II algorithm is used to obtain the rank and crowding distance, which are used for selection. The proposed algorithm was compared with an ARX model corresponding to a second-order system.

The model was used to obtain both the 10-step ahead and the 20-step ahead predictor and the normalized mean square error. The R^2^ statistics are also presented. The results show that the model is capable of prediction with varying parameters that may be encountered during everyday operation. We noticed that in both the simulation model and the experimental model the predictor did not take the mass of the payload into account. This can be attributed to the high mechanical impedance of the crane in the experimental stand and *M* >> *m* in the simulated model.

Future work includes using a larger payload mass in the simulation and experiments and reducing the complexity of the proposed algorithm. The model obtained will also be used in designing the controller, as well as an online parameter estimator that would minimize both transient and residual sway as well as minimizing the time it takes to move from one point to another in the workspace.

## Figures and Tables

**Table 1:** Simulation model parameters.

Parameters	Values
*M* [kg]	500
*m* [kg]	10–50
*l* [m]	0.5–3
$f_{fr0}$	4.4
ξ	0.01
$k_{p}$	0.05
$k_{r}$	0.45

**Table 2:** Multi-gene genetic programming parameters for simulated data.

Parameters	Simulation
Population size	200
Number of generations	100
Initialization method	PTC2 [31]
Max tree depth during initialization	5
Max number of genes	8
Terminal set	*u*(k−1), …, *u*(k−5), *y*(k−1), …, *y*(k−3), *m*, *g*, 1/*l*, *t_s_*
Non-terminal set	+, −, ×, analytic quotient
High level crossover	0.2
Mutation	0.14
Tournament size	8
Prediction horizon	20

**Table 3:** Gene weights for the position and sway predictor.

Weights	*x*(*k*)	*α*(*k*)
*θ*_1_	−0.9947	−0.9987
*θ*_2_	1.9947	−0.1042
*θ*_3_	−8.1689 × 10^−6^	−1.0723 × 10^−5^
*θ*_4_	1.3829 × 10^−4^	1.9993
*θ*_5_	2.6866 × 10^−5^	−8.4135 × 10^−6^
*θ*_6_	−2.9573 × 10^−8^	−1.5966 × 10^−4^

**Table 4:** Coefficients of the simulation position and sway ARX models.

Coefficients	*x*(*k*)	*α*(*k*)
*b*_1_	1.0229 × 10^−5^	−5.9573 × 10^−6^
*b*_2_	9.4147 × 10^−6^	−6.1440 × 10^−6^
*a*_1_	1.9944	1.8967
*a*_2_	−0.9944	−0.9914

**Table 5:** Statistics of the identified prediction model of the simulated system.

Output	Parameters	NMSE				R^2^			
		10-Step Ahead		20-Step Ahead		10-Step Ahead		20-Step Ahead	
		MGGP	ARX	MGGP	ARX	MGGP	ARX	MGGP	ARX
Position	*l* = 0.9 m; *m* = 10 kg	0.0013	0.0028	0.0046	0.0043	0.9999	0.9998	0.9995	0.9996
	*l* = 0.9 m; *m* = 40 kg	0.0016	0.0028	0.0047	0.0043	0.9999	0.9998	0.9994	0.9996
	*l* = 1.3 m; *m* = 40 kg	0.0017	0.0029	0.0050	0.0046	0.9999	0.9998	0.9994	0.9995
	*l* = 1.7 m; *m* = 10 kg	0.0013	0.0028	0.0047	0.0044	0.9999	0.9998	0.9995	0.9996
Sway	*l* = 0.9 m; *m* = 10 kg	0.0578	0.2840	0.0776	0.3823	0.9868	0.6988	0.9762	0.4702
	*l* = 0.9 m; *m* = 40 kg	0.0577	0.3226	0.0320	0.4466	0.9866	0.6136	0.9958	0.2662
	*l* = 1.3 m; *m* = 40 kg	0.0572	0.1082	0.0380	0.1562	0.9868	0.9568	0.9941	0.9126
	*l* = 1.7 m; *m* = 10 kg	0.0618	0.2402	0.0595	0.5006	0.9850	0.7876	0.9862	0.0787

**Table 6:** Multi-gene genetic programming parameters for experimental data.

Parameters	Simulation
Population size	200
Number of generations	100
Initialization method	PTC2 [31]
Max tree depth during initialization	5
Max number of genes	8
Terminal set	*u*(k−5), …, *u*(k−9), *y*(k−1), …, *y*(k−3), *m*, *g*, 1/*l*, *t_s_*
Non-terminal set	+, −, ×, analytic quotient
High level crossover	0.2
Mutation	0.14
Tournament size	8
Prediction horizon	20

**Table 7:** Gene weights for the position and sway predictor.

Weights	*x*(*k*)	*α*(*k*)
*θ*_1_	0.1025	−0.0753
*θ*_2_	−0.1025	1.4887
*θ*_3_	−1.2238 × 10^−4^	−0.5138
*θ*_4_	−0.0062	9.0125 × 10^−4^
*θ*_5_	1.6469	1.0233 × 10^−5^
*θ*_6_	-	−0.0010

**Table 8:** Coefficients of the experimental position and sway ARX models.

Coefficients	*x*(*k*)	*α*(*k*)
*b*_1_	0.0031	−2.1083 × 10^−4^
*b*_2_	0.0423	8.2345 × 10^−5^
*a*_1_	1.8249	1.8980
*a*_2_	−0.8249	−0.9626

**Table 9:** Statistics of the identified prediction model of experimental stand.

Output	Parameters	NMSE				R^2^			
		10-Step Ahead		20-Step Ahead		10-Step Ahead		20-Step Ahead	
		MGGP	ARX	MGGP	ARX	MGGP	ARX	MGGP	ARX
Position	*l* = 0.9 m; *m* = 10 kg	0.0109	0.0214	0.0169	0.0320	0.9964	0.9876	0.9891	0.9662
	*l* = 0.9 m; *m* = 40 kg	0.0113	0.0229	0.0169	0.0353	0.9962	0.9861	0.9895	0.9600
	*l* = 1.3 m; *m* = 40 kg	0.0115	0.0218	0.0154	0.0330	0.9959	0.9869	0.9907	0.9634
	*l* = 1.7 m; *m* = 10 kg	0.0119	0.0235	0.0177	0.0353	0.9959	0.9857	0.9888	0.9615
Sway	*l* = 0.9 m; *m* = 10 kg	0.0799	0.2851	0.0893	0.3898	0.9753	0.7384	0.9640	0.5694
	*l* = 0.9 m; *m* = 40 kg	0.1450	0.3355	0.1599	0.4245	0.9185	0.6375	0.8771	0.4721
	*l* = 1.3 m; *m* = 40 kg	0.1722	0.3416	0.1770	0.2925	0.8726	0.7235	0.7911	0.6647
	*l* = 1.7 m; *m* = 10 kg	0.1911	0.3487	0.1815	0.4868	0.8404	0.6222	0.7561	0.3685

## Data Availability

The data presented in this study are available upon request from the corresponding author.
